# Isolation and Characterization of Extracellular Vesicles from *Arabidopsis thaliana* Cell Culture and Investigation of the Specificities of Their Biogenesis

**DOI:** 10.3390/plants12203604

**Published:** 2023-10-18

**Authors:** Yulia Yugay, Zhargalma Tsydeneshieva, Tatiana Rusapetova, Olga Grischenko, Anastasia Mironova, Dmitry Bulgakov, Vladimir Silant’ev, Galina Tchernoded, Victor Bulgakov, Yury Shkryl

**Affiliations:** 1 Federal Scientific Center of the East Asia Terrestrial Biodiversity of the Far East Branch of Russian Academy of Sciences, Vladivostok 690022, Russia; zargalma2509@gmail.com (Z.T.); avramenko.dvo@gmail.com (T.R.); crab_ol@mail.ru (O.G.); mistletoe8@gmail.com (A.M.); bulgakov-dv@mail.ru (D.B.); tchernoded@biosoil.ru (G.T.); bulgakov@biosoil.ru (V.B.); 2 School of Medicine and Life Sciences, Far Eastern Federal University, Vladivostok 690922, Russia; vladimir.silantyev@gmail.com; 3 Institute of Chemistry, Far Eastern Branch of Russian Academy of Sciences, Vladivostok 690022, Russia

**Keywords:** exosomes, nanoparticles, membrane trafficking, callus culture, salicylic acid

## Abstract

Over recent years, extracellular vesicles (EVs), commonly termed exosomes, have gained prominence for their potential as natural nanocarriers. It has now been recognized that plants also secrete EVs. Despite this discovery, knowledge about EV biogenesis in plant cell cultures remains limited. In our study, we have isolated and meticulously characterized EVs from the callus culture of the model plant, *Arabidopsis thaliana*. Our findings indicate that the abundance of EVs in calli was less than that in the plant’s apoplastic fluid. This difference was associated with the transcriptional downregulation of the endosomal sorting complex required for transport (ESCRT) genes in the calli cells. While salicylic acid increased the expression of ESCRT components, it did not enhance EV production. Notably, EVs from calli contained proteins essential for cell wall biogenesis and defense mechanisms, as well as microRNAs consistent with those found in intact plants. This suggests that plant cell cultures could serve as a feasible source of EVs that reflect the characteristics of the parent plant species. However, further research is essential to determine the optimal conditions for efficient EV production in these cultured cells.

## 1. Introduction

Extracellular membrane vesicles, also known as exosomes, are widely distributed in various biological systems [1]. Our understanding of exosomes has primarily been focused on their presence in mammals [2]. However, over the past decade, there has been a surge in scientific interest in investigating similar nano-sized membrane structures derived from plant species. In plants, extracellular vesicles (EVs) were first described as secretory vesicles from carrot cells cultured in auxin-containing media [3]. The existence of EVs has been discerned in the apoplastic fluid of leaves and roots [4,5], as well as in plant fruits and freshly extracted juices derived from them [6,7]. The mechanisms underlying the formation of plant EVs remain incompletely elucidated despite their similarities to the exosome biogenesis pathway employed by animal cells [8]. It is hypothesized that EVs are formed through three distinct mechanisms, resulting in a diverse range of fractions: the multivesicular body pathway (MVB), the exocyst-positive organelle pathway (EXPO), and the vacuolar pathway [8,9]. The MVB pathway is crucial for EV production in plants [10].

Exosome biogenesis is intricately linked to intracellular membrane trafficking and commences with the formation of an early endosome through the inward folding of the plasma membrane in accordance with the regulatory constituents of the Golgi trans-network [11]. The early endosome undergoes maturation through endosomal membrane invagination and subsequent intraluminal vesicles (ILVs) generation. The maturation process produces MVBs and facilitates the accumulation of diverse biomolecules [12]. Subsequently, MVBs fuse with the plasma membrane, allowing EVs to be released into the extracellular environment [13]. In mammals, this mechanism is regulated by a distinct endosomal sorting complex required for transport (ESCRT) [11]. The ESCRT molecular machinery comprises four distinct protein complexes, namely, ESCRT-0, ESCRT-I, ESCRT-II, and ESCRT-III, accompanied by various accessory components [14]. Briefly, this intricate biological mechanism exhibits the ability to identify proteins that have undergone ubiquitination and subsequently guide them toward vesicle formation. The vesicles were subsequently internalized by MVB. Marker signals of this biological process include the presence of specific proteins such as tetraspanin 8 (TET8) [15,16,17], secretory syntaxin penetration 1 PEN1 [18,19], and heat-shock protein 70 (HSP70) [20,21], which are enriched within EVs.

Similar to the exosomes found in mammals, plant EVs are responsible for transporting various biomolecules and facilitating communication between different cells, species, and across kingdoms [8]. They play a role in regulating developmental processes, activating the immune system, and facilitating the development of a protective response to stressors [22,23,24,25,26]. In general, EVs exhibit a composition akin to that of exosomes found in mammals, including microRNAs (miRNAs), proteins, and secondary metabolites [23,24,25,26]. In contrast to mammalian exosomes, plant-derived EVs exhibit non-immunogenic properties, and their large-scale generation is economically more efficient [27]. Several plant exosomes are undergoing clinical trials as components for various therapeutic agents. Lemon exosomes, identified as constituents of a patented dietary supplement [28], contain cardiovascular agents present in the supplement. Exosomes derived from ginger roots, including unmodified and curcumin-loaded variants, are currently being evaluated for their therapeutic potential in bowel disease. The ongoing clinical trials registered under the identifiers NCT01294072 and NCT04879810 are aimed at investigating the efficacy of these exosomes [29,30].

Plant cell cultures also serve as viable reservoirs of EVs. Plant cell cultures possess certain advantages over plants in terms of their capacity for scalability and stability of growth parameters. Only a limited number of studies have successfully isolated exosomes from plant cell cultures or their corresponding culture media. EVs have been isolated from callus and suspension cultures of *Aster yomena* [31], *Nicotiana tabacum* [32], and *Craterostigma plantagineum* [26]. However, the molecular mechanisms governing EVs biogenesis within plant cell cultures remain unexplored.

In this study, we performed a comparative analysis of EVs sourced from *Arabidopsis thaliana* cell cultures and the apoplastic fluid of the plant. Our aim was to identify similarities and differences in their qualitative and quantitative characteristics, as well as in their biomolecular composition, specifically focusing on miRNAs and proteins. To gain a deeper understanding of EVs biogenesis in callus cells, we analyzed the expression of the core components of the ESCRT complex and the plasma membrane-associated proteins TET8 and PEN1, comparing their levels in callus cells with those in intact plants. Additionally, we investigated the impact of the stress hormone salicylic acid, on EVs biogenesis in the plant cell culture.

## 2. Results and Discussion

### 2.1. Isolation and Characterization of Arabidopsis EVs

**Figure 1:**
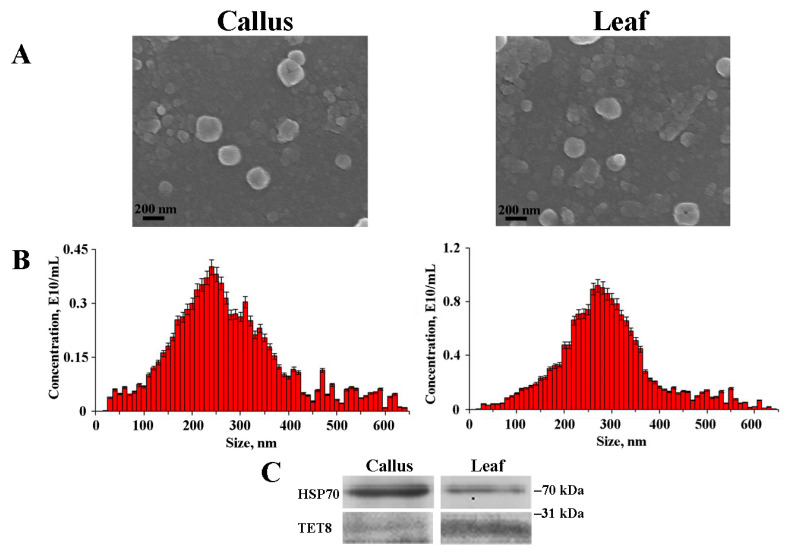
Scanning electron microscopy images (**A**) and nanoparticle tracking analysis (**B**) of EVs isolated from *A. thaliana* calli and leaf apoplastic fluid. The presence of HSP70 and TET8 proteins in EVs are shown in a Western blot (**C**).

EVs were isolated from the callus and leaf apoplastic fluid of *A. thaliana* through ultracentrifugation. SEM analysis revealed that the size and structural integrity of the EVs remained consistent across various samples. Notably, SEM images showed that the diameter of the EVs ranged from approximately 90 to 250 nm while also displaying an imperfectly spherical shape (Figure 1A). This particular morphology can be attributed to the presence of proteins within the dense lipid membrane, a phenomenon that has been elucidated in earlier studies [33,34]. Interestingly, a comparable morphological structure was observed for the nanoparticles derived from ginger [35]. NTA was used to determine the size distribution of EVs isolated from *Arabidopsis*. This analysis revealed a heterogeneous population of particles of various sizes. The calculated average sizes of the isolated EVs were 222.8 nm and 283.6 nm for calli and apoplastic fluid, respectively (Figure 1B, Table 1). The striking similarity in dimensions is evident from the size measurements of 209.3 nm attributed to tobacco callus EVs, determined through NTA, as reported in a study by Kocholata et al. [32], and the approximate size of 225.2 nm observed for *Aster yomena* callus-derived EVs, obtained via dynamic light scattering (DLS) analysis [31]. This cross-species uniformity suggests the existence of regulatory mechanisms governing EV biogenesis. The presence of TET8 and HSP70 proteins was monitored by Western blotting before starting the experiments (Figure 1C and Figure S1). Previously, the presence of HSP70 [36] and TET8 [15,37] confirmed the nature of EVs.

The zeta (Z) potential values of the EVs were analyzed using NTA, and the results are presented in Table 1. The evaluation of surface charge revealed that EVs derived from both *A. thaliana* calli and leaf apoplastic fluid exhibited a strong negative Z-potential. The average values were quantified as −23.8 mV for calli-derived EVs and −30.5 mV for those obtained from leaf apoplast. The negative surface charge of *Arabidopsis* EVs indicates their potential to interact with positively charged molecules or surfaces, thereby facilitating specific cellular uptake or adhesion events. The presence of negatively charged EVs has been previously documented in both plant and animal preparations [38,39]. The negative charge, essential for the colloidal stability of EVs, is commonly attributed to phosphate moieties present on the vesicle surfaces [40]. An intriguing observation is that the negative charge on EVs can foster preferential adhesion to inflammatory regions [40], analogous to the behavior exhibited by negatively charged liposomes [41]. The net negative charge of EVs also renders them amenable to purification methods utilizing polycations, such as protamine and polyethylene glycol [42].

NTA was also used to quantify the concentration of EVs in the studied biological samples. To calculate the yield, the vesicle concentration was determined relative to 1 g of either callus or plant biomass. For *A. thaliana* calli and leaf apoplastic fluids, EV concentrations were measured at 1.8 × 10^10^ and 2.9 × 10^10^ EVs g^−1^ FW, respectively (Table 1). Although the difference in content by 1.6 times is not overly pronounced, the results obtained suggest a slightly reduced potential of cultured cells to produce EVs. Interestingly, it has been reported that the concentration of EVs isolated from tobacco calli was notably lower, measuring 0.057 × 10^9^ EVs g^−1^ FW [32]. The marked difference in vesicle concentration between tobacco callus culture and our findings may arise from variations in the timing of callus harvest and the proliferation rate of the calli. It is essential to note that quantifying EV concentration in plants and calli should be interpreted with caution, as it might not precisely represent the overall biological pattern. The secretion of EVs is influenced by the physiological state of cells and is integral to the intercellular trafficking of diverse signaling molecules [43]. Therefore, variations in the concentration of EVs could be attributed to differences in cellular states during the harvest period, underscoring the dynamic nature of EV generation in response to the cellular growth phase. Nevertheless, we can reasonably expect reduced EV production in calli due to their undifferentiated state and optimal growth conditions. Callus cells may not require heightened levels of active communication or pathogen defense, which likely translates to a dampened EVs biogenesis. The intriguing interplay between the cellular differentiation status and exosome production underscores the regulatory nature of vesicle secretion in response to distinct physiological needs. Further investigations and additional comparative experiments on other plant models at different growth phases are necessary to fully elucidate the underlying mechanism of this phenomenon.

### 2.2. miRNA Accumulation in Arabidopsis EVs

**Figure 2:**
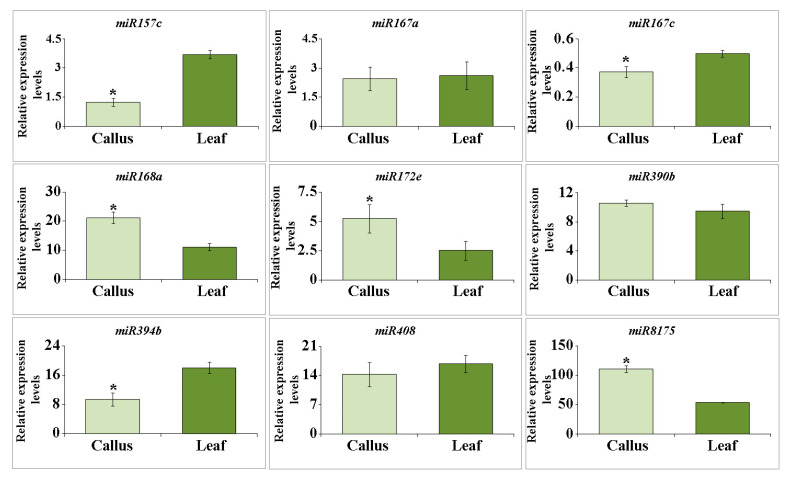
Expression levels of exosomal miRNA in EVs isolated from *A. thaliana* calli and leaf apoplastic fluid measured by real-time PCR in three independent biological replicates. Data shown are mean ± SE, n = 3. Asterisks denote significant differences at *p* < 0.05 (*), Student’s *t*-test.

The abundance of small RNA patterns encompassing miRNAs and tiny RNAs within plant EVs is a recognized phenomenon [44]. In this study, we focused on the selection of miRNA species to determine their presence within EVs from *Arabidopsis* callus cultures. This subset includes four key miRNA variants, miR167c, miR390b, miR394b, and miR408, all prominently identified in the secreted fraction of *A. thaliana* EVs [45]. Additionally, our analysis included five representative miRNA species: miR157c, miR167a, miR168a, miR172e, and miR8175. These miRNA species are expected to reside predominantly within the confines of EVs [46]. The expression levels of miR157e, miR167c, miR394b, and miR408 were 2.9-, 3.5-, 1.9 and 1.2 times higher in plant EVs than in calli (Figure 2). However, the abundances of miR168a, miR172e, and miR8175 transcripts were 1.9-, 2 and 2 times higher in callus EVs than in plants, respectively. The expression levels of miR167a, miR390b, and miR408 did not differ significantly. Among the miRNAs examined, miR8175, miR168a, miR394b, miR408, miR390b, and miR172e were the predominant constituents, comprising a major fraction of the miRNA pool. Slightly lower levels were observed for miR157e and miR167a reflecting a distinct distribution pattern within the samples. Interestingly, miR167c transcripts displayed notably lower levels of accumulation than other miRNAs. It was previously revealed that *Arabidopsis* EVs encompass various RNA-binding proteins, such as argonaute 1 (AGO1), helicases, and annexins [47]. Remarkably, AGO1 has emerged as the sole member secreted by nanovesicles that engages specifically with exosomal miRNAs rather than their cellular counterparts. Conversely, annexins play a role in nonspecific binding to miRNAs, contributing significantly to their stabilization within the exosomal environment.

Collectively, our findings elucidate the miRNA composition of calli-derived EVs, revealing a distinct pattern that aligns with that of typical *Arabidopsis* EVs. However, this accumulation varies across different miRNA species. This observation prompted us to consider the existence of discrete sorting mechanisms that govern miRNA loading into EVs in both plant and cell culture systems. Whether this selective accumulation is influenced by the miRNA sequence, duplex structure, target specificity, or other functional attributes remains to be determined.

### 2.3. Protein Composition of Callus-Derived EVs

The capacity of EVs to accumulate a diverse repertoire of proteins is well established [43]. We analyzed the protein composition of purified EVs derived from *Arabidopsis* callus, revealing proteins associated with hormone signaling, responses to various stresses, cell wall organization, and other key cellular functions, as detailed in Table 2 and Table S2. Data suggest that the protein composition of vesicles is not random, as many of these proteins are consistently identified in EVs across various plants and sources. A notable finding was the presence of the GDSL esterase/lipase EPITHIOSPECIFIER MODIFIER1 (ESM1) within the vesicles. Members of the GDSL family play pivotal roles in diverse plant cell functions, encompassing stress responses, secondary metabolism, morphogenesis, development, and growth [48,49,50,51,52]. Specifically, the ESM1 protein is known to alter glucosinolate hydrolysis, leading to the accumulation of toxic isothiocyanate and heightened resistance to plant feeders [53,54]. The presence of ESM1 in callus-derived vesicles suggests the retention of plant exosome loading mechanisms and potentially underscores their involvement in glucosinolate biosynthesis in callus cultures. Moreover, our analysis identified proteins, including heat shock 70 kDa protein, myrosinase 2, vegetative storage protein 1, polygalacturonase inhibitor 1, and beta-glucosidase isoforms, that are associated with defense and immune responses to both biotic and abiotic stresses [55,56,57]. Our findings are consistent with those of EVs originating from *Arabidopsis* apoplastic fluid. These vesicles, enriched with defense proteins, exhibit heightened release in response to *Pseudomonas syringae* infection and salicylic acid treatment [18]. In addition, we identified several UDP-arabinopyranose mutase isoforms, probable xyloglucan endotransglucosylase/hydrolase 11, and alpha-xylosidase 1. All of these proteins are associated with cell wall organization [58,59,60,61,62,63]. This finding is consistent with earlier observations in *Arabidopsis* suspension cultures, where these proteins play a role in cell wall regeneration [64]. Interestingly, hydrolases involved in cell wall remodeling are also enriched in EVs isolated from *C. plantagineum* and *N. tabacum* suspension cultures [26]. This suggests the potential contribution of EVs to cell wall expansion and construction, particularly in the context of actively dividing callus cells.

### 2.4. Expression of Genes Participating in EVs Biogenesis

**Figure 3:**
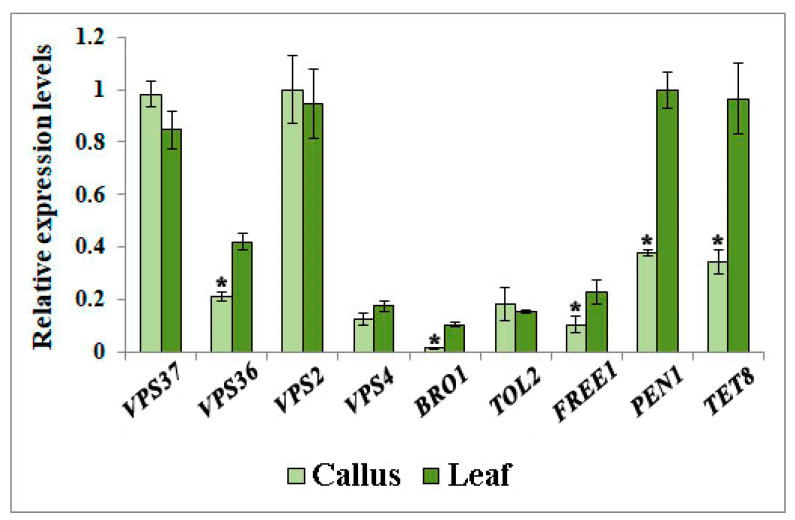
Expression levels of genes participating in EVs biogenesis in *A. thaliana* callus and leaf samples measured by real-time PCR in three independent biological replicates. Data shown are mean ± SE, n = 3. Asterisks denote significant differences at *p* < 0.05 (*), Student’s *t*-test.

EVs biogenesis is a complex process tightly orchestrated by a specialized endosomal sorting complex required for transport (ESCRT). In plants, this complex is composed of four distinct protein components, namely ESCRT-I, -II, and -III, along with vacuolar protein sorting 4 (VPS4) ATPase [14]. In our study, we observed that the expression levels of *VPS37* (ESCRT-I subunit) and *VPS2* (ESCRT-III subunit) were consistent in the calli and leaves of *A. thaliana* (Figure 3). Conversely, the expression of *VPS36* (ESCRT-II subunit) and plant-specific FREE1 (FYVE DOMAIN PROTEIN REQUIRED FOR ENDOSOMAL SORTING 1), which is incorporated into the ESCRT-III complex [69], in *Arabidopsis* leaves was twice that in calli. The transcription level of BRO1-like domain-containing protein 1 (BRO1) was 8 times lower in callus cultures than in plants. BRO1 plays a significant role in ESCRT function. It interacts with other ESCRT components to facilitate the recognition and sequestration of ubiquitinated cargo into intraluminal vesicles and aids in the scission of vesicles from the limiting membranes of MVBs [70]. Additionally, the expression levels of *VPS4* in callus and plant samples were nearly identical. As an AAA-ATPase, VPS4 is necessary to dissociate and recycle ESCRT-III components [71]. Similarly, no significant differences were detected in the expression of genes encoding TOL2 (TOM1-like), an upstream ESCRT factor that partially fulfills the ESCRT-0 function in plants [72]. The coordinated interaction between ESCRT complexes and related proteins plays a pivotal role in vesicle biogenesis, where ESCRT-I initiates assembly, ESCRT-II aids in membrane deformation, and ESCRT-III finalizes scission [14]. Our findings suggest that the down-regulation of specific components within the ESCRT complex, such as VPS36, BRO1, and FREE1, may disrupt, to a certain extent, the sorting of cargo and/or the formation of EVs in callus culture.

In our examination of EVs biogenesis in *A. thaliana* cultured cells, we also explored the expression levels of TETRASPANIN 8 (TET8) and PENETRATION 1 (PEN1) in both plant and callus samples (Figure 3). These plasma membrane-associated proteins are involved in EV biogenesis and are classified into distinct plant EV classes [16,17,18,47]. Our analysis revealed that the transcript counts of *PEN1* and *TET8* were significantly higher in plants than in calli, with 2.5-fold and 2.7-fold increases, respectively (Figure 2). It has been previously shown that *A. thaliana*’s response to invasion by the phytopathogenic fungus *Botrytis cinerea* triggers considerable upregulation of *TET8* gene expression, leading to the subsequent accumulation of defense proteins within EVs [47]. In support of this observation, both single *tet8* and *tet8*/*tet9* double mutants in *Arabidopsis* showed decreased EV quantities and increased susceptibility to *B. cinerea* infection [47]. This underlines TET8’s potential role in enhancing the immune response of plants against infections by facilitating EV formation. Additionally, PEN1, a syntaxin, has been implicated in fusion between the plasma membrane and secretory trafficking vesicles [73]. Despite this established function, the involvement of MVB in this process remains unclear and requires further investigation [74]. Collectively, our findings indicate that the expression of several components involved in EV biogenesis is inhibited in *Arabidopsis* calli, thus shedding light on their poor EV productivity.

### 2.5. The Effect of Salicylic Acid on EVs Biogenesis

**Figure 4:**
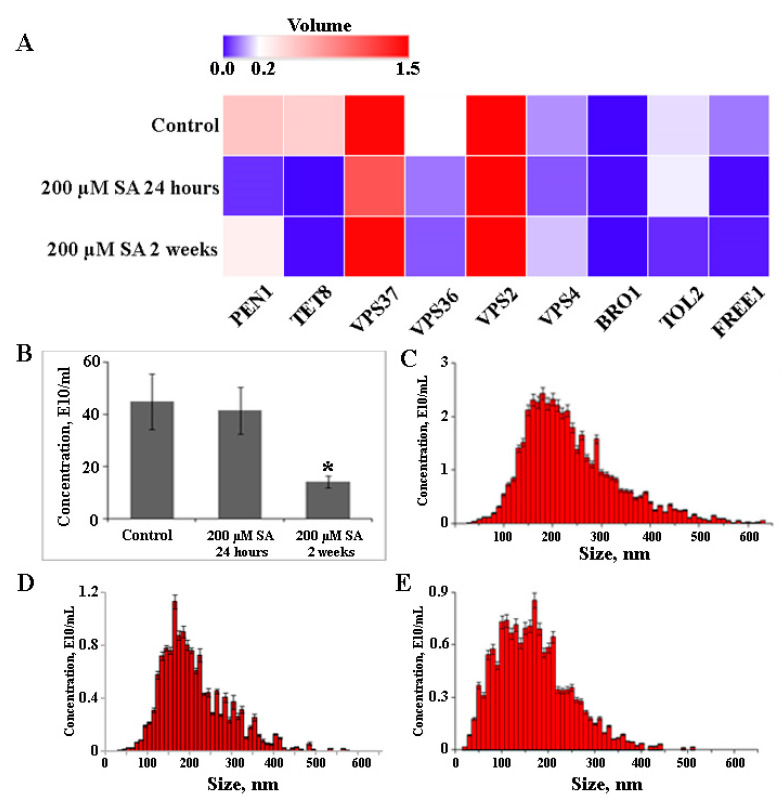
Expression levels of genes involved in EV biogenesis (**A**) and EV concentrations (**B**) in *A. thaliana* callus under control and salicylic acid (SA) treatments. Nanoparticle tracking analysis of EVs isolated from A. thaliana control calli (**C**) compared to those from SA-treated calli after 24 h (**D**) and 2 weeks (**E**). Data shown are mean ± SE, n = 3. Asterisks denote significant differences at *p* < 0.05 (*), Student’s *t*-test.

Salicylic acid (SA) is recognized as a key signaling molecule in plant defense responses to various biotic and abiotic stresses [75]. Given the association between vesicle biogenesis and plant stress responses, we hypothesized that emulating such conditions could stimulate EV secretion in vitro. Moreover, SA treatment increased EV secretion in *A. thaliana* leaves [18]. The callus culture was exposed to 200 µM SA for 24 h or two weeks to emulate stress conditions, and the subsequent impact on EV production and related gene expression was assessed. After a 2-week exposure to SA, *FREE1* and *PEN1* expression levels increased by 1.6- and 5.2-fold, respectively, but no significant changes were noted after a 24-h treatment compared to control calli (Figure 4A). In contrast, 24 h post-SA treatment, the expression levels of *VPS36*, *VPS37*, and *VPS2* increased by 4-, 7-, and 8-fold respectively, and this effect persisted with prolonged exposure. Transcriptional activity of *VPS4* and *TOL2* genes exhibited contrasting dynamics in response to SA. *VPS4* expression rose with extended SA incubation, marking a 5-fold increase after 24 h and 10-fold after 2 weeks compared to the control. *TOL2* expression increased 9-fold after 24 h, but this boost receded to a mere 1.5-fold difference from the control after 2 weeks. Interestingly, SA treatment facilitated the transcriptional activation of genes like *PEN1*, *VPS36*, and *FREE1*. These genes had been notably downregulated in calli but returned to levels comparable to those in plants. In contrast, *TET8* expression, already lowered in calli, declined further by 13-fold and 6-fold under short-term and long-term SA exposure, respectively. A substantial 6-fold increase in *BRO1* transcriptional activity was observed 24 h after SA treatment; however, after 2 weeks, its level decreased by 1.3 times compared to the control. Interestingly, the concentration of EVs remained stable in callus treated with SA for 24 h (Figure 4B). The average sizes of EVs from control and SA-treated cells were comparable, measuring 233.6 ± 28.1 nm and 204.63 ± 23.7 nm, respectively (Figure 4C,D). In contrast, long-term SA-treated cells showed an almost threefold reduction in EV content compared to untreated cells (Figure 4B), along with a decrease in the average EV size to 163.5 ± 19.2 nm (Figure 4E). Notably, this specific SA dose did not affect the accumulation of A. thaliana callus biomass compared to the control conditions during long-term treatment. These findings highlight the complex interplay between SA-induced activation and EV formation in plants and calli. Moreover, the long-term SA treatment could induce other indirect or non-defense-related effects [76], which mask the effect of the hormone on EV biogenesis. The observed differential responses suggest that the ESCRT machinery requires precise regulation for accurate cargo sorting and vesicle formation. Gaining insights into these delicate mechanisms will enhance our understanding of plant cell biology and could offer practical applications in modulating vesicle-mediated processes in cultured plant cells.

## 3. Materials and Methods

### 3.1. Plant Material and Growth Conditions

For this study, we selected the *Arabidopsis thaliana* ecotype Columbia-0 (Col-0) as the plant material. *Arabidopsis* seeds were cultivated side-by-side in soil under controlled conditions. The growth environment was maintained at 22 °C with a photoperiod of 16 h of light followed by 8 h of darkness. The *A. thaliana* callus culture was previously established using Col-0 seedlings [77]. The calli were cultivated on a solid medium supplemented with 0.5 mg/L of 2,4-dichlorophenoxyacetic acid and 2.5 mg/L of 6-benzylaminopurine. The calli were housed in 50-mL glass Erlenmeyer flasks and maintained at 25 °C in the dark. To induce a salicylic acid (SA) response, calli were cultivated in the presence of 200 µM concentration of the hormone for two weeks.

### 3.2. Isolation of EVs

EVs were isolated from freshly harvested rosette leaves of 6-week-old *A. thaliana* in accordance with a commonly applied protocol [78]. In brief, leaves were infiltrated twice with potassium phosphate-buffered saline (PBS); pH 7.4 (Bio-Rad Laboratories, Hercules, CA, USA) using a Concentrator plus (Eppendorf, Hamburg, Germany). After infiltration, the excess buffer on the leaf surface was removed using paper napkins. To isolate the apoplastic fluid, infiltrated leaves were placed in 30 mL needle-free syringes. These syringes were then positioned in 50 mL tubes and centrifuged at 700× *g*. To eliminate cellular contaminants and subcellular structures a series of centrifugation steps were employed, involving successive spins at 5000× *g*, 10,000× *g*, and 20,000× *g* for 30 min at 4 °C. The resulting supernatant was passed through a 0.45 μm nylon membrane filter. The filtrate was transferred to ultracentrifuge tubes and spun at 100,000× *g* for 1 h at 4 °C using an Optima MAX-XP ultracentrifuge with a TLA 110 rotor (Beckman Coulter, Brea, CA, USA). To extract EVs from *Arabidopsis* calli, we used freshly harvested cells from two-week-old cultures. These cells underwent three PBS washes to remove the residual nutrient medium. Post-wash, surplus liquid was discarded by filtration through a grid, and the cells were homogenized with a mixer. The subsequent steps mirrored those described for plants. EVs precipitates obtained from both the plant apoplastic fluid and callus culture were washed twice with PBS and finally resuspended in 1 mL of PBS.

### 3.3. Scanning Electron Microscopy

The morphology of the purified EVs was characterized using scanning electron microscopy (SEM). Prior to analysis, the EVs were fixed in 4% glutaraldehyde for 20 min. The EVs were washed twice with PBS and spotted onto carbon-coated grids. The droplets were then air-dried. SEM images were obtained using a field emission scanning electron microscope (FE-SEM) Sigma (Carl Zeiss, Jena, Germany). Observations were carried out at the accelerating voltage of 10 kV and vacuum of 10^−5^ mm of mercury. The «InLens» detector was used during the investigations.

### 3.4. Nanoparticle Tracking Analysis

The hydrodynamic diameters of the extracted EVs were determined using nanoparticle tracking analysis (NTA) with a Nanosight NS500 system (Malvern Instruments, Malvern, UK), following the manufacturer’s guidelines. Before analysis, samples were diluted with water to achieve an approximate count of 20–50 particles per frame for particle size measurements, and 100–200 particles per frame for zeta potential assessment. For particle size measurements, the autofocus was adjusted to ensure particle clarity. Ten videos of particle motion resulting from Brownian motion were recorded for each sample, each lasting 60 s, at a temperature of 23 °C. For the zeta potential assessment, the manufacturer’s integrated automated algorithm was used. During each measurement, three cycles were executed. Within each cycle, 11 cell positions were scanned, capturing 60 frames per position. The video setting was on “high,” with the following specific settings: focus set to autofocus, camera sensitivity at 92.0, shutter speed at 70, scattering intensity at 4.0, and a cell temperature of 23 °C. The recorded video data were subsequently processed using NTA analysis software version 2.2.

### 3.5. Isolation of RNA and miRNA, cDNA Synthesis, and PCR Analysis

Total RNA was isolated from both plant and callus samples using the phenol-guanidine method with the ExtractRNA reagent (Evrogen, Moscow, Russia). For gene expression analysis, 1 µg of total RNA was reverse transcribed in a 20 µL reaction volume using the RNAscribe RT Reverse Transcriptase kit (Biolabmix, Novosibirsk, Russia) following the manufacturer’s recommendations. The resulting cDNA was stored at −20 °C or used immediately for quantitative real-time PCR (qPCR). Gene-specific primer pairs targeting *A. thaliana* genes, including *TET8, PEN1, VPS37-1, VPS36, VPS2-1*, *VPS4*, *BRO1*, *TOL2*, and *FYVE1* (Supplementary Table S1) were used. *GAPDH* was used as the reference gene for normalization.

To isolate miRNA from plant and calli EVs, they underwent pre-treatment with 5 mg/mL Proteinase K for 10 min at 37 °C. Subsequently, 5 mM phenylmethylsulfonyl fluoride (PMSF; Sigma-Aldrich, Saint Louis, MO, USA) was added, and the mixture was incubated for an additional 10 min at room temperature. Proteinase K was inactivated by heating the samples at 90 °C for 5 min. The EVs were then exposed to RNase A (final concentration 0.5 µg/µL, Thermo Fisher Scientific, Waltham, MA, USA) for 20 min at 37 °C. The extraction of miRNAs was carried out utilizing the «Lira» Kit for microRNA isolation (Biolabmix, Novosibirsk, Russia). Meticulous on-column DNase digestion was performed to eliminate any potential DNA contamination. Following extraction, miRNA concentration was quantified using a microRNA assay kit (Thermo Fisher Scientific, Waltham, MA, USA) on a Fluo-100 A fluorometer (Allsheng, Hangzhou, China). miRNA species within vesicles derived from callus and apoplastic fluid of *A. thaliana* were evaluated using the stem-loop method described by Yang et al. [79]. A universal stem-loop primer was integrated into the reverse transcription reaction in place of the oligo(dT)15 primer typically used for cDNA synthesis. Other parameters remained as described above. For qPCR, a universal reverse primer was paired with forward primers specific to each miRNA (Supplementary Table S1).

Three biological replicates sourced from distinct RNA or miRNA extractions were analyzed. Three technical replicates were analyzed for each biological replicate. qPCR was performed using HS-qPCR 2x SYBR Blue Master Mix (Biolabmix, Novosibirsk, Russia) on a CFX96 Real-Time System (Bio-Rad Laboratories) following previously established methodologies [80,81]. The expression levels were normalized using the 2^−ΔΔCT^ approach [82], with transcript amounts measured in relation to the sample expressing the highest level. CFX Manager Software (Version 1.5; Bio-Rad Laboratories) was used for data analysis.

### 3.6. Isolation and Analysis of Protein Cargo

#### 3.6.1. Extraction Procedure

The EVs derived from calli were treated with Proteinase K (20 µg/mL) for 1 h at 37 °C, with intermittent gentle mixing every 10 min to ensure uniform exposure of the vesicles to the enzyme. Following Proteinase K treatment, the proteolytic activity was curtailed by introducing 5% PMSF into the samples. The subsequent inhibition process involved incubation for 10 min at room temperature. Further inactivation of the proteinase was achieved by subjecting the samples to a heat treatment at 90 °C for 5 min. The extraction of total proteins followed established protocols, as previously described [77]. Protein extracts obtained from the EV samples were separated by 12.5% SDS-PAGE and visualized by Coomassie staining. Upon completion of electrophoresis, gel slices containing the protein components of interest were meticulously excised into smaller fragments. The excised gel slices were subjected to in-gel digestion with trypsin (Trypsin V511; Promega, Madison, WI, USA) using a previously described procedure [83]. Following trypsin digestion, the resulting peptide mixture was dried using a Concentrator plus (Eppendorf, Hamburg, Germany).

#### 3.6.2. Mass Spectrometry and Protein Identification

Peptide mass spectra were acquired using an Autoflex MALDI-TOF mass spectrometer (Bruker Daltonics, Bremen, Germany) with a nitrogen laser operating in positive reflector mode. A standard method RP 700-3500 Da.par was employed to capture the mass spectra, ensuring comprehensive coverage of the desired mass range. For instrument control, FlexControl software (version 3.4; Bruker Daltonics, Bremen, Germany) was employed, using a nitrogen laser in automatic mode (AutoXecute—automatic Run) for analysis. External calibration of the spectra was achieved using CalibratePeptide Standards, FAMS Method, and a standard calibration mixture (Protein Calibration Standard I, Bruker Daltonics, Bremen, Germany). Subsequently, the Flexanalysis program version 3.4 (Bruker Daltonics, Bremen, Germany) was used for automated peak extraction. A SNAP signal detection program (Bruker Daltonics, Bremen, Germany) was used to automatically assign the first monoisotopic signals in the spectra. Specific methods were utilized for both MS and MS/MS analyses: PMF.FAMSMethod for MS and SNAP_full_process for MS/MS. Each spectrum was generated by averaging 1500–5000 laser shots acquired at the minimum laser power (300 shots per step).

The obtained data were analyzed using the BioTools software (version 3.2; Bruker Daltonics, Bremen, Germany). Database searches were conducted using the Mascot search engine and m/z spectra against the *A. thaliana* NCBInr and SwissProt databases. A peptide mass tolerance of 0.5 Da and a fragment mass tolerance of 0.5 Da were employed for database matching. A threshold score of 40 was used to ensure reliable identification during the database search. The mass spectrometry proteomics data have been deposited to the ProteomeXchange Consortium via the PRIDE [84] partner repository with the dataset identifier PXD045849 and 10.6019/PXD045849

#### 3.6.3. Western Blotting

Proteins isolated from callus-derived ELVs were Western blotted using rabbit polyclonal antibodies against TET8 and HSP70 as primary antibodies (PHY1490S and PHY0167, respectively, PhytoAB, San Jose, CA, USA). Immunoreactive bands were detected with the anti-rabbit goat IgG conjugated with alkaline phosphatase (ab97048, Abcam, Cambridge, UK) followed by CDP-Star™ Substrate (T2146, Thermo Fisher Scientific, Waltham, MA, USA) detection reagent as described previously [85].

#### 3.6.4. Statistical Analysis

All values are expressed as the mean ± SE. For statistical evaluation, the Student’s *t*-test was used to compare the two independent groups. The level of statistical significance was set at *p* < 0.05.

## 4. Conclusions

In this study, we analyzed the biogenesis of EVs in *A. thaliana* callus cultures and contrasted this with the EVs produced by intact plants. Our analyses demonstrated that the EVs from callus cultures are morphologically and dimensionally consistent with those sourced from leaf apoplastic fluid. Particle sizes, as determined by SEM, ranged between 90 and 250 nm. However, NTA, which assesses the hydrodynamic radius influenced by particle surface properties, reported sizes of 222.8 nm for callus-derived EVs and 283.6 nm for those from apoplastic fluid. Notably, these measurements align with reported EV dimensions from various plant sources when analogous methodologies were employed. Furthermore, both callus-derived and leaf apoplast-derived EVs displayed a significant negative zeta potential, suggesting enhanced colloidal stability and potential implications for their biological interactions.

Proteomic analyses indicated that *Arabidopsis* callus-derived EVs are enriched in proteins associated primarily with stress responses and cell wall modifications. This protein signature closely mirrors that of EVs isolated from other plants and cell cultures. Additionally, these callus-derived EVs contained specific miRNAs found in EVs from apoplastic fluid, although the abundance of certain miRNAs varied. It is noteworthy that the callus-derived EV yield was inferior to that of intact plant tissues. Moreover, while a 24-h treatment with the plant hormone salicylic acid had no discernible effect on EV yield, a two-week exposure resulted in a marked reduction.

Transcriptional profiles highlighted differential expression patterns of key ESCRT complex proteins and associated factors, including PEN1 and TET8, which might influence EV biogenesis and cargo specificity. A significant transcriptional downregulation was observed in ESCRT-II, ESCRT-III components, and the BRO1 factor. SA treatment intensified the transcription of ESCRT-II and ESCRT-III components across both short and prolonged exposures, whereas BRO1 and TET8 transcripts were further reduced. Based on these findings, we hypothesize that the dedifferentiated state of callus cultures, combined with their specific culture conditions, might inherently reduce the requirement for EV-mediated intercellular communication.

Given the alignment of EV production from plant cell cultures with the stringent quality metrics of the pharmaceutical industry [86,87], there is a compelling case for a more granular exploration of the molecular underpinnings of EV biogenesis in these cell cultures. Such endeavors can catalyze the formulation of strategic frameworks for leveraging EVs in diverse applications.

## Figures and Tables

**Table 1:** Characteristics of EVs isolated from *A. thaliana* calli and apoplastic fluid.

Samples	Average Size, nm	Ζ-Potential, mV	Concentration, Particles/g FW *
Calli	222.8 ± 36.5	−23.8 ± 1.3	1.8 × 10^10^
Leaf apoplastic fluid	283.6 ± 58.3	−30.5 ± 2.2	2.9 × 10^10^

* FW, fresh weight. Data shown are mean ± SE, n = 3.

**Table 2:** Protein composition of EVs isolated from callus culture of *A. thaliana*.

Name of Protein	Protein Function	Occurrence in Plant EVs
Stress responses		
Heat shock 70 kDa protein (HSP70)	Folds *de novo* synthesized proteins and protect cells from heat stress conditions.	*A. thaliana* leaf apoplastic fluid [18], *C. plantagineum* in vitro cultured cells [26], juices from ginger [30], citrus [65], tomato [66], broccoli [67], and grape [68]
GDSL esterase/lipase (ESM1)	Regulation of biotic and abiotic stress responses. Possesses antimicrobial and antifungal activities.	*A. thaliana* leaf apoplastic fluid [18], *C. plantagineum* in vitro cultured cells [26], and juice from ginger [30]
Myrosinase 2 (BGL37)	Involved in the abscisic acid signaling pathway and has redundant functions in glucosinolate breakdown and insect defense.	Juice from ginger [30]
Germin-like protein subfamily 3 member 1 (GL31)	Manganese ion binding. May play a role in plant defense.	-
Vegetative storage protein 1 (VSP1)	Involved in the jasmonic acid signaling pathway and plays a role in defense against herbivorous insects.	*A. thaliana* leaf apoplastic fluid [18]
Polygalacturonase inhibitor 1 (PGIP1)	Inhibits the pectin-depolymerizing activity of polygalacturonases secreted by microbial pathogens and insects.	*C. plantagineum* in vitro cultured cells [26], juices from ginger [30] and citrus [65]
CCR4-associated factor 1 homolog 4 (CAF1D)	Shows mRNA deadenylation activity and plays a role in plant defense responses to pathogen infections.	-
Cell wall remodeling		
UDP-arabinopyranose mutases 1–4 (RGP1, RGP2, RGP3, and RGP4)	Converts UDP-arabinopyranose to UDP-arabinofuranose for cell wall and natural product biosynthesis; also plays a role in the pollen development process.	*C. plantagineum* in vitro cultured cells [26], juices from citrus [65], tomato [66], and broccoli [67]
Xyloglucan endotransglucosylase/hydrolase protein 11 (XTH11)	Participates in cell wall biogenesis, organization, and the xyloglucan metabolic process.	Juices from ginger [30], *C. plantagineum* in vitro cultured cells [26], and tomato [66]
Alpha-xylosidase 1 (XYL1)	Regulation and maintenance of cell wall physical properties.	*C. plantagineum* in vitro cultured cells [26] and juice from ginger [30]
Beta-glucosidases 22 and 23 (BGL22 and BGL23)	Facilitates the hydrolysis of terminal, non-reducing beta-D-glucosyl residues, resulting in the release of beta-D-glucose. It also plays a role in plant protection by releasing scopoletin and other active compounds under both biotic and abiotic stress conditions.	*A. thaliana* leaf apoplastic fluid [18], *C. plantagineum* in vitro cultured cells [26], juices from ginger [30], citrus [65], and tomato [66]
Various metabolic pathways		
Sucrose synthase 4 (SUS4)	Sucrose-cleaving enzyme that provides UDP-glucose and fructose for various metabolic pathways.	*C. plantagineum* in vitro cultured cells [26], juices from ginger [30], citrus [65], and tomato [66]
Beta carbonic anhydrase 1 (BCA1)	Facilitates the reversible hydration of carbon dioxide, essential for photosynthesis in cotyledons, and is involved in the CO_2_ signaling pathway.	*A. thaliana* leaf apoplastic fluid [18], juices from ginger [30], citrus [65], and tomato [66]
Phosphoglycerate kinase 3 (PGKY3)	Catalyzes reactions in glycolysis and plays a crucial role in carbohydrate metabolism.	*A. thaliana* leaf apoplastic fluid [18], *C. plantagineum* in vitro cultured cells [26], juices from ginger [30], citrus [65], and grape [68]
Glyceraldehyde-3-phosphate dehydrogenase GAPC1 (G3PC1)	Participates in the photosynthetic reductive pentose phosphate pathway, crucial for maintaining cellular ATP levels and carbohydrate metabolism.	*A. thaliana* leaf apoplastic fluid [18], *C. plantagineum* in vitro cultured cells [26], juices from ginger [30], citrus [65], and tomato [66]
Nitrilase 1 and 2 (NRL1 and NRL2)	Plays a key role in the biosynthesis of the plant hormone auxin. It also aids in detoxifying harmful nitriles.	*A. thaliana* leaf apoplastic fluid [18], juices from ginger [30] and citrus [65]
EP1-like glycoprotein 3 (EP1L3)	A protein from the curculin-like lectin family that may be involved in a cell-to-cell programmed cell death (PCD) signaling mechanism.	*A. thaliana* leaf apoplastic fluid [18], *C. plantagineum* in vitro cultured cells [26], and juice from ginger [30]

## Data Availability

Data are available in the article, Supplementary Materials, or by request to the corresponding authors.

## Supplementary Materials

The following supporting information can be downloaded at: https://www.mdpi.com/article/10.3390/plants12203604/s1, Table S1. List of primers used in this study. Table S2. Protein composition of EVs isolated from callus culture of *A. thaliana*. Figure S1. Western blot analysis of EVs isolated from *A. thaliana* callus culture and apoplast washing fluid. Proteins (40 µg) were resolved using a 10% SDS-PAGE gel. EVs marker proteins detected were: (A) HSP70 (71/72 kDa) and (B) TET8 (31 kDa).
