# Supplementary material for: Isolation and Characterization of Extracellular Vesicles from Arabidopsis thaliana Cell Culture and Investigation of the Specificities of Their Biogenesis
Source: Plants (Basel). 2023 Oct 18;12(20):3604. doi: 10.3390/plants12203604 (PMC10609744; doi:10.3390/plants12203604)
Supplement: Supplementary file 1 [file plants-12-03604-s001.zip › Supplementary Materials.pdf]

Supplementary Table S1. List of primers used in this study.

Gene (GenBank accession no.)	Forward (5′–3′)	Reverse (5′–3′)
GAPDH (AT1G13440)	TTGGTGACAACAGGTCAAGC	AACTTGTCGCTCAATGCAATC
TET8 (AT2G23810)	ACTTGGTGATTACTCTACTTG	AGGTTTGGCAGTCTGGATTA
PEN1 (AT3G11820)	TCGTCCGAGTATAGAGAAAC	AGCACGGCCATGTCTAGAA
Universal stem-loop primer	GAAAGAAGGCGAGGAGCAGATCGAGGAAGAAGACGGAAGAATGTGCGTCTCGCCTTCTTTCNNNNNNNN	
Universal reverse primer	-	CGAGGAAGAAGAAGACGGAAGAAT
miR157c (AT3G18217)	CGAGGAAGAAGAAGACGGAAG	-
miR167a (AT3G22886)	TTGAAGCTGCCAGCATGATC	-
miR167c (AT3G04765)	TTGAAGCTGCCAGCATGATC	-
miR168a (AT4G19395)	TCGCTTGGTGCAGGTCGG	-
miR172e (AT5G59505)	ATGGAATCTTGATGATGCTGC	-
miR390b (AT5G58465)	CGAAGCTCAGGAGGGATAG	-
miR394b (AT1G76135)	ATTGGCATTCTGTCCACCTC	-
miR408 (AT2G47015)	ATGCACTGCCTCTTCCCTG	-
miR8175 (AT2G05455)	GATCCCCGGCAACGGCC	-
VPS37-1 (At3g53120)	ATTCTACTCCCCTGGTTCTC	CGTCGATGATAGGTAGTACG
VPS36 (AT5G04920)	AGGAACATCTACTATCTGCAG	CCGTATGTCCCAAACTCCTT
VPS2-1 (AT2G06530)	GTATCGACATCAACCAAGAG	TCTATACCTCCACTGTCCTC
VPS4 (AT4G26750)	CCTTCTCACTACCAAAACCC	TGATGAGTAAGGTGGAGGAG
BRO1 (AT1G15130)	TGAGGGCCTGAAGTTCTATG	TATGGGCCGGAGCTTCTATG
TOL2 (AT1G06210)	GAAAGCCAAATCACACAGAG	TCAGATGCAGAGCCTCGAAC
FREE1 (AT1G20110)	GTTTGAGGGAAGGATCTGGG	TCAATGTGCGCTAACGAGGA

A

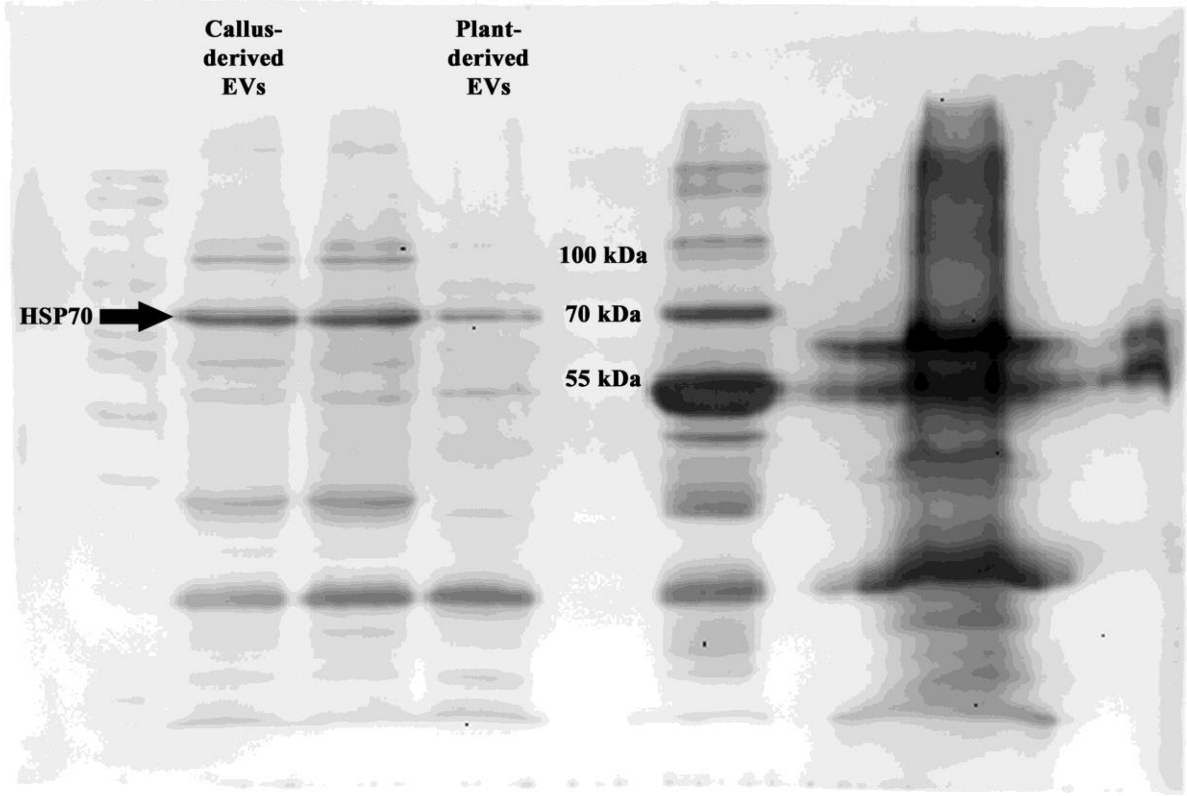

B

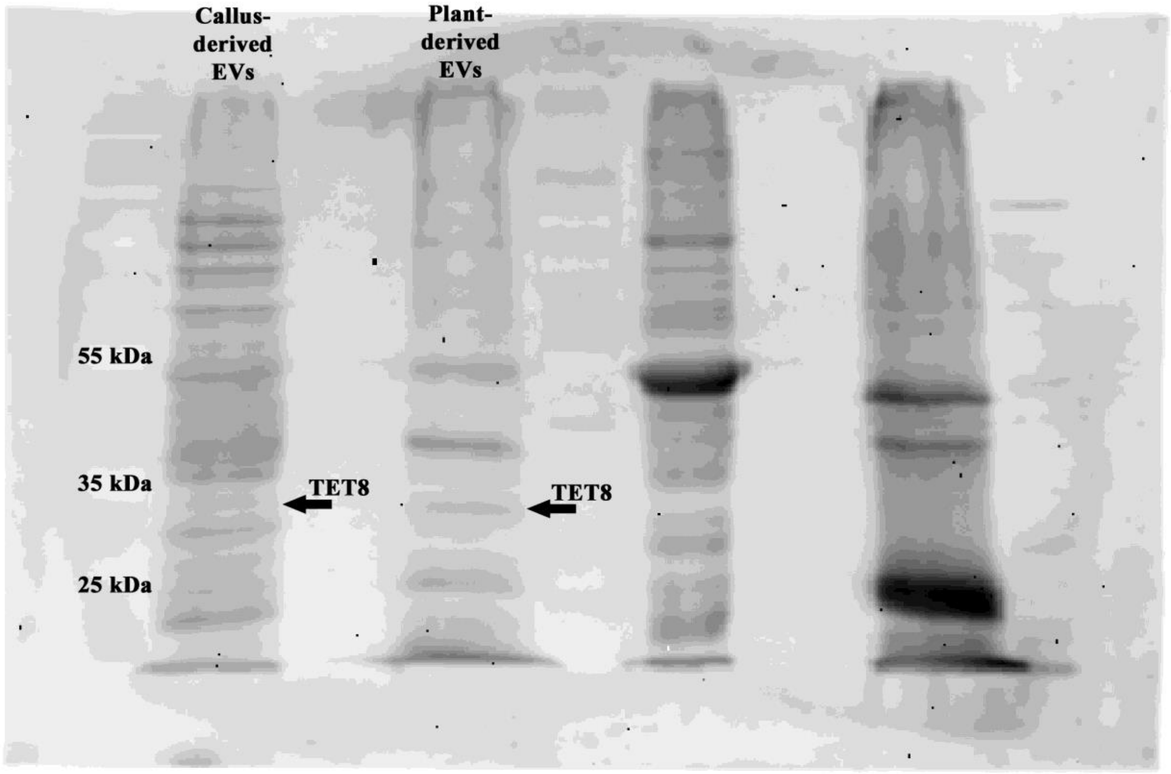

Supplementary Figure S1. Western blot analysis of EVs isolated from *A. thaliana* callus culture and apoplast washing fluid. Proteins (40 µg) were resolved using a 10% SDS-PAGE gel. EVs marker proteins detected were: (**A**) HSP70 (71/72 kDa) and (**B**) TET8 (31 kDa).

Supplementary Table S2. Protein composition of EVs isolated from callus culture of *A. thaliana*.

Name of protein	Score	Mass	Peptides	Unique peptides	Observed mass/sequences
Heat shock 70 kDa protein (HSP70)	159	72946	8	6	1417.6941 [K.EVDEVLLVGGMTR.V]; 1453.6907 [R.EAELHAQKDKER.K]; 1550.7545 [K.AVVTVPAYFNDAQR.Q]; 1581.7806 [R.EAELHAQKDKERK.E]; 1613.8314 [R.SRFETLVNHLIER.T]; 1821.8857 [K.SQVFSTAADNQTQVGIR.V]
GDSL esterase/lipase (ESM1)	89	44032	6	6	1038.4021 [R.SYFFFDGR.H]; 1367.6017 [K.DLPQTYWPYGK.S]; 1408.6409 [R.ELIVYPTGETMR.E]; 1794.9683 [K.FVVQLLAPLGCLPIVR.Q] + Carbamidomethyl (C); 2100.9444 [K.ANPNADASAQQAFVTNVINR.L]; 2570.1685 [K.AQEEMAHLLYGADPDVVQPMTVR.E]
Myrosinase 2 (BGL37)	39	62692	4	4	1301.5325 [R.GLNVWDGFTHR.Y]; 1301.6966 [R.GLNVWDGFTHR.Y]; 1327.7294 [K.ASGLWYQSFLR.D]; 2153.1903 [R.GINEDGINYYSGLIDGLIAR.N]
Germin-like protein subfamily 3 member 1 (GL31)	42	21545	4	4	1436.3863 [A.SVQDFCVANLKR.A]; 1799.8546 [R.AETPAGYPCIRPIHVK.A] + [+47.9847 at C9]; 1808.8865 [R.AETPAGYPCIRPIHVK.A]; 1964.9722 [K.RAETPAGYPCIRPIHVK.A]
Vegetative storage protein 1 (VSP1)	60	30243	3	2	1536.7026 [R.VFKLPNPLYYVPS]; 1388.9514 [K.GYNIVGNIGDQWADLVEDTPGR.V]; 2517.0282 [K.KGYNIVGNIGDQWADLVEDTPGR.V]
Beta carbonic anhydrase 1 (BCA1)	57	37426	1	1	1906.1378 [R.EAVNVSLANLLTYPFVR.E]
UDP-arabinopyranose mutase 1 (RGP1)	140	40602	14	4	1178.5557 [K.TGLPYIYHSK.A]; 1444.5257 [R.YDDMWAGWCIK.V] + [+57.0215 at C9]; 1460.5184 [R.YDDMWAGWCIK.V] + [+72.9952 at I10]; 1606.6586 [K.YIFTIDDDCFVAK.D] + [+57.0215 at C9]; 1729.6889 [K.GTLFPMCGMNLAFDR.E] + [+57.0215 at M6]; 1742.7338 [K.GTLFPMCGMNLAFDR.E] + [+70.0419 at C7]; 1745.6852 [K.GTLFPMCGMNLAFDR.E] + [+72.9952 at L3]; 1786.7728 [K.TIAVPEGFDYELYNR.N]; 1925.8777 [K.GIFWQEDIIPFFQSAK.L]; 2121.9427 [R.ELIGPAMYFGLMGDGQPIGR.Y]; 2137.9395 [R.ELIGPAMYFGLMGDGQPIGR.Y] + [+15.9949 at Y8]; 2365.0337 [K.NLLCPSTPFFFNTLYDPYR.E; + [+57.0215 at C4]; 3035.4605 [R.EGVSTAVSHGLWLNIPDYDAPTQLVKP

					K.E]; 3217.6721 [M.VEPANTVGIPVNHIPLLKDELDIVIPTIR.N] + [+42.0470 at K18]
UDP-arabinopyranose mutase 2 (RGP2)	53	40864	13	5	1178.5557 [K.TGLPYIYHSK.A]; 1444.5257 [R.YDDMWAGWCIK.V] + [+57.0215 at C9]; 1460.5184 [R.YDDMWAGWCIK.V] + [+72.9952 at I10]; 1606.6586 [K.YIFTIDDDCFVAK.D] + [+57.0215 at C9]; 1729.6889 [K.GTLFPMCGMNLAFDR.E] + [+57.0215 at M6]; 1742.7338 [K.GTLFPMCGMNLAFDR]; 1745.6852 [K.GTLFPMCGMNLAFDR.D] + [+72.9952 at L3]; 1767.7332 [K.IHVPEGYDYELYNR.N]; 1895.8272 [K.KIHVPEGYDYELYNR.N]; 2107.9247 [R.DLIGPAMYFGLMGDGQPIGR.Y]; 2137.9395 [R.DLIGPAMYFGLMGDGQPIGR.Y] + [+29.9742 at Y8]; 2351,0200 [K.NLLCPSSPFFFNTLYDPYR.E] + [+57.0215 at N-term N]; 3035.4605 [R.EGVSTAVSHGLWLNIPDYDAPTQLVKPK.E]
UDP-ara binopyranose mutase 3 (RGP3)	96	41254	7	2	1444.5257 [R.YDDMWAGWCVK.V; + [+71.0371 at C9]; 1460.5184 [R.YDDMWAGWCVK.V] + [+87.0143 at C-term K]; 1729.6889 [K.GTLFPMCGMNLAFDR.E] + [+57.0215 at M6]; 1742.7338 [K.GTLFPMCGMNLAFDR.E] + [+70.0419 at C7]; 1745.6852 [K.GTLFPMCGMNLAFDR.E] + [+72.9952 at L3]; 2121.9427 [R.ELIGPAMYFGLMGDGQPIGR.Y]; 2137.9395 [R.ELIGPAMYFGLMGDGQPIGR.Y] + [+15.9949 at Y8]
EP1-like glycoprotein 3 (EP1L3)	61	49020	2	2	960.5236 [R.WVWEANR.G]; 2287.1975 [R.VVNEGGYTDYSPIEYNPDVR.G]
Nitrilase 1 (NRL1)	90	38128	3	3	1603.8037 [K.YHASAIHVPGPEVAR.L]; 1821.9265 [K.GAELVLFPEGFIGGYPR.G]
Nitrilase 2 (NRL2)	52	37130	1	1	1837.9168 [K.GSELVVFPEAFIGGYPR.G]
Polygalacturonase inhibitor 1 (PGIP1)	113	36666	3	3	1078.3897 [K.TTWSIDLSR.N]; 1649.5307 [K.LQTFDSYSYFHNK.C]; 2076.8239 [K.LTGSIPESFGSFPGTVPDLR.L]
Phosphoglycerate kinase 3 (PGKY3)	28	42105	1	1	1465.6484 [K.FLKPSVAGFLMQK.E]
Probable xyloglucan endotransglucosylase/hydrolase protein 11 (XTH11)	36	31919	1	1	2182.1109 [K.NSGSGFESQLIYGSGYFNVR.I]
Alpha-xylosidase 1	41	10233	3	3	1376.6194 [K.TVSWWGDEIKR.F]; 1471.6440

(XYL1)					[R.WIEVGAFYPFSR.D]; 1681.6702 [R.QELYQWDTVADSAR.N]
Beta-glucosidase 22 (BGL22)	27	59743	6	4	1714.6510 [R.CSGHNADVAVDFFHR.Y] + Pyro-carbamidomethyl (N-term C); 1731.6862 [R.CSGHNADVAVDFFHR.Y] + [+57.0215 at S2]; 2689.2577 [R.SGYEAYLVSHNLLNAHAEAVEVFR.Q]; 2888.3468 [K.IGIAHSPAWFEPHDLKDSNDVPTVSR.V]
Putative CCR4-associated factor 1 homolog 4 (CAF1D)	35	34840	2	2	1075.4448 [K.IAESCSGLSSR.F]; 1237.5222 [K.KIAESCSGLSSR.F]
Beta-glucosidase 23 (BGL23)	82	59683	6	6	950.6664 [K.NLNTDAFR.M]; 1250.8489 [R.GPALWDIYCR.R] + Carbamidomethyl (C); 2251.4879 [K.NAQNYAIGSKPLTAALNVYSR.G]; 2706.6729 [R.SGYEAYLVTHNLLISHAEAVEAYR.K]; 2834.7679 [R.SGYEAYLVTHNLLISHAEAVEAYRK.C]; 3169.8278 [K.ASTDFVGLNYYTSVFSNHLEKPDPSKPR.W]
Probable UDP-arabinopyranose mutase 4 (RGP4)	37	41839	2	2	1767.8936 [K.IRVPEGYDYELYNR.N] + [-19.0422 at R2]; 1786.9365 [K.IRVPEGYDYELYNR.N]
Sucrose synthase 4 (SUS4)	59	92944	7	7	1021.6264 [R.WISSQMNR.V]; 1043.6218 [K.GGAFFEFLR.S]; 1053.6574 [R.SFTLPGLYR.V]; 1267.8453 [R.IKQQGLNITPR.I]; 1282.7649 [K.YIGDGVEFLNR.H]; 1463.9367 [R.IQNLNTLQHNLR.K]; 1539.0409 [R.LRELVNLVVVGGDR.R]
Glyceraldehyde-3-phosphate dehydrogenase GAPC1 (G3PC1)	25	36891	2	2	[K.LVSWYDNEWGYSSR.V]; [K.GILGYTEDDVVSTDFVGDNR.S]
